# Assessing the concurrent validity of a gait analysis system integrated into a smart walker in older adults with gait impairments

**DOI:** 10.1177/0269215519852143

**Published:** 2019-05-27

**Authors:** Christian Werner, Georgia Chalvatzaki, Xanthi S Papageorgiou, Costas S Tzafestas, Jürgen M Bauer, Klaus Hauer

**Affiliations:** 1 Centre for Geriatric Medicine, Heidelberg University, Heidelberg, Germany; 2 Agaplesion Bethanien Hospital Heidelberg, Geriatric Centre at the Heidelberg University, Heidelberg, Germany; 3 School of Electrical and Computer Engineering, National Technical University of Athens, Athens, Greece

**Keywords:** Smart walker, gait analysis, elderly, validity, assistive devices

## Abstract

**Objective:**

To assess the concurrent validity of a smart walker–integrated gait analysis system with the GAITRite^®^ system for measuring spatiotemporal gait parameters in potential users of the smart walker.

**Design:**

Criterion standard validation study.

**Setting:**

Research laboratory in a geriatric hospital.

**Participants:**

Twenty-five older adults (⩾65 years) with gait impairments (habitual rollator use and/or gait speed <0.6 m/s) and no severe cognitive impairment (Mini-Mental State Examination ⩾17).

**Main measures:**

Stride, swing and stance time; stride length; and gait speed were simultaneously recorded using the smart walker–integrated gait analysis system and the GAITRite system while participants walked along a 7.8-m walkway with the smart walker. Concurrent criterion-related validity was assessed using the Bland–Altman method, percentage errors (acceptable if <30%), and intraclass correlation coefficients for consistency (ICC_3,1_) and absolute agreement (ICC_2,1_).

**Results:**

Bias for stride, swing and stance time ranged from −0.04 to 0.04 seconds, with acceptable percentage errors (8.7%–23.0%). Stride length and gait speed showed higher bias (mean_bias_ (SD) = 0.20 (0.11) m; 0.19 (0.13) m/s) and not acceptable percentage errors (31.3%–42.3%). Limits of agreement were considerably narrower for temporal than for spatial-related gait parameters. All gait parameters showed good-to-excellent consistency (ICC_3,1_ = 0.72–0.97). Absolute agreement was good-to-excellent for temporal (ICC_2,1_ = 0.72–0.97) but only poor-to-fair for spatial-related gait parameters (ICC_2,1_ = 0.37–0.52).

**Conclusion:**

The smart walker–integrated gait analysis system has good concurrent validity with the GAITRite system for measuring temporal but not spatial-related gait parameters in potential end-users of the smart walker. Stride length and gait speed can be measured with good consistency, but with only limited absolute accuracy.

## Introduction

Recent technological developments in gait analysis focus on ambulatory solutions that allow for unobtrusive and continuous gait monitoring in real-life environments outside the laboratory such as wearable sensors.^1^ However, these sensors require an individual’s willingness to wear them and may cause discomfort and adherence issues. In addition, to our knowledge, the validity of body-worn sensors for measuring spatiotemporal gait parameters in older adults with walking aids is still unknown. Considering that walkers or rollators are prescribed routinely to patients during geriatric rehabilitation and that many older adults with mobility limitations have to use them for ambulation, there is the need for valid gait analysis systems to unobtrusively and continuously capture spatiotemporal gait parameters also in these walking aid users.

Technological advances have led to the development of ‘smart walkers’ with various high-tech functionalities such as monitoring a user’s gait.^2^ Different sensor types (e.g. vision-based sensors,^3^ inertial measurement units,^4^ force sensors^5^) have been used to implement gait analysis on a smart walker. Independent of the technical implementations, to our knowledge, previous validation studies of smart walker–integrated gait analysis systems suffered from methodological shortcomings such as small sample sizes, participants not representative of potential users, no criterion standard comparisons and/or no statistical analyses.^3–5^

The study aim was to assess the concurrent validity of a smart walker–integrated gait analysis for measuring spatiotemporal gait parameters with a criterion standard (GAITRite^®^ system) in a reasonable number of potential smart walker users.

## Methods

The study was conducted between 1 November and 5 December 2014, with approval of the ethics committee of the Medical Faculty of the Heidelberg University (S-358/2013) and in accordance with the Declaration of Helsinki. All participants gave written informed consent.

Participants were recruited from rehabilitation wards of a geriatric hospital, from nursing homes and from a hospital-associated sports club for geriatric outpatient rehabilitation. According to the defined users of our smart walker,^6^ inclusion criteria were age ⩾65 years, moderate gait impairments (rollator use in daily life and/or 4 m usual gait speed^7^ <0.6 m/s) and no severe cognitive impairment (Mini-Mental State Examination^8^ score ⩾17 points).

The GAITRite system (CIR Systems Inc., Havertown, PA, USA) is an electronic walkway with embedded pressure sensors, representing a well-established and validated method for automated gait analysis in clinical settings.^9^ The GAITRite system used in this study was 5.79 m long and 0.89 m wide (active area: 4.88 m × 0.61 m; sampling rate 120 Hz).

The smart walker integrates innovative functionalities such as sit-to-stand assistance, obstacle avoidance, navigation assistance and gait monitoring. A detailed description of all its functionalities has been provided previously.^6,10^ For this study, only the gait analysis system of the smart walker was activated and all other innovative functionalities were deactivated. The smart walker–integrated gait analysis system is based on a standard laser range finder (UBG-04LX-F01; Hokuyo Automatic Co., Ltd, Osaka, Japan; sampling period 28 ms/scan) mounted at the rear side of the four-wheeled smart walker at a fixed height of 35 cm from the ground with a viewing direction towards the user’s legs to record their motion at a horizontal plane below the knee level. Gait parameters were extracted by pre-processing the laser data using a Probabilistic Data Association Particle Filtering system and subsequent modelling of the user’s walking pattern based on a Hidden Markov Model approach, as previously described.^11^ The overall goal of this gait analysis system is not only to validly measure gait parameters continuously during smart walker use but also to serve as a basis for future development of a context-aware smart walker that generates and provides real-time assistive actions (e.g. distance/velocity adjustments) according to the user‘s current walking pattern.

After a familiarization phase, in which participants freely moved around with the smart walker for approximately 2–5 minutes, they were instructed to walk along a GAITRite instrumented walkway with the smart walker at self-selected maximum gait speed. Each walk was initiated and terminated 1 m before and after the walkway (total length = 7.79 m) to account for acceleration and deceleration. No practice trials were performed on the instrumented walkway. After data recording, each walk was checked to ensure that the same steps, and the same number of steps, were used to calculate mean values for spatiotemporal gait parameters (stride, swing and stance time; stride length; and gait speed) by both processing methods. Mean values were used because average gait parameters are usually of clinical interest.

Between-method differences (bias) and 95% limits of agreement (mean_bias_ ± 1.96 × SD_bias_) were determined using the Bland–Altman method.^12^ Percentage errors, calculated as 100 × (1.96 × SD_bias_)/((mean_smart walker_ + mean_GAITRite_)/2), were considered to be clinically acceptable if <30%.^13^ Intraclass correlation coefficients (ICCs) with 95% confidence intervals were calculated to determine the consistency (ICC_3,1_) and absolute agreement (ICC_2,1_) between the mean gait parameters measured by the two methods. ICCs were interpreted as poor (<0.40), fair to good (0.40–0.75) and excellent (>0.75).^14^ The sample size for this study was estimated to be ⩾23 participants, based on an acceptable ICC of 0.70 and an expected ICC of 0.90 for two measurements (smart walker and GAITRite), a significance level (α) of 0.05 and a statistical power (1–β) of 0.80.^15^ A two-sided *P*-value of <0.05 indicated statistical significance. Statistical analysis was performed using IBM SPSS Statistics for Windows, Version 25.0 (IBM Corp., Armonk, NY, USA).

## Results

The sample included 25 older adults with a mean (SD) age of 84.1 (5.4) years, moderate gait impairments (usual gait speed = 0.48 (0.15) m/s) and no severe cognitive impairment (Mini-Mental State Examination score = 24.5 (4.1) points). Sixteen (64%) participants were geriatric rehabilitation patients, seven (28%) were members of the sports club for geriatric outpatient rehabilitation, and two (8%) were nursing home residents.

**Figure 1.**
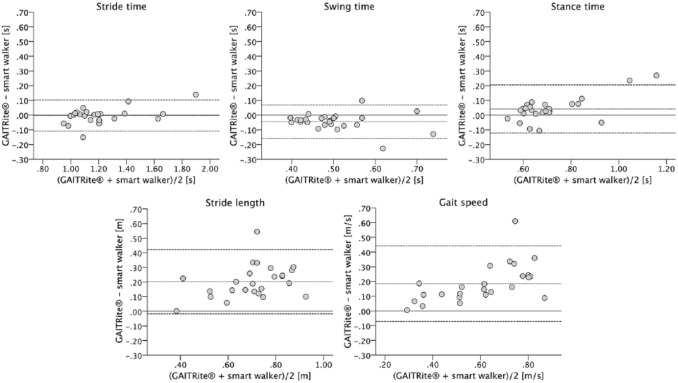
Bland–Altman plots for comparison between the GAITRite^®^ system and the smart walker–integrated gait analysis system. Dotted lines indicate bias and dashed lines indicate upper and lower 95% limits of agreement (±1.96 SD of the bias).

Mean bias for the stride, swing and stance time ranged from –0.04 to 0.04 seconds, with clinically acceptable percentage errors (8.7%–23.0%) (Table 1). Stride length and gait speed showed both a substantially higher bias (mean_bias_ (SD) = 0.20 (0.11) m; 0.19 (0.13) m/s) and a clinically not acceptable percentage error (31.3%–42.3%). Limits of agreement were considerably narrower for the stride (–0.10 to 0.11 seconds), swing (–0.07 to 0.16 seconds) and stance time (–0.12 to 0.20 seconds) than for the stride length (–0.07 to 0.44 m) and gait speed (–0.02 to 0.42 m/s) (Figure 1). Consistency between both methods was good to excellent for all gait parameters (ICC_3,1_ = 0.72–0.97). Absolute agreement was also good to excellent for the stride, swing and stance time (ICC_2,1_ = 0.72–0.97), but only poor to fair for the stride length (ICC_2,1_ = 0.37) and gait speed (ICC_2,1_ = 0.52).

**Table 1.** Mean values (±SD), mean difference scores (bias ± SD), limits of agreement, mean percentage errors and intraclass correlation coefficients for consistency and absolute agreement for each gait parameter.

Gait parameter	GAITRite^®^	Smart walker	Bias	95% LOA	PE	Consistency ICC_3,1_ (95% CI)	Absolute agreement ICC_2,1_ (95% CI)
Stride time (s)	1.21 ± 0.24	1.21 ± 0.22	–0.01 ± 0.05	–0.10 to 0.11	8.7	0.97 (0.94 to 0.99)	0.97 (0.94 to 0.98)
Swing time (s)	0.48 ± 0.09	0.52 ± 0.10	–0.04 ± 0.06	–0.07 to 0.16	22.8	0.80 (0.59 to 0.91)	0.72 (0.27 to 0.89)
Stance time (s)	0.73 ± 0.18	0.69 ± 0.13	0.04 ± 0.08	–0.12 to 0.20	23.0	0.86 (0.70 to 0.93)	0.83 (0.62 to 0.93)
Stride length (m)	0.80 ± 0.17	0.60 ± 0.13	0.20 ± 0.11	–0.02 to 0.42	31.3	0.72 (0.45 to 0.86)	0.37 (–0.10 to 0.73)
Gait speed (m/s)	0.70 ± 0.22	0.51 ± 0.15	0.19 ± 0.13	–0.07 to 0.44	42.3	0.76 (0.53 to 0.89)	0.52 (–0.10 to 0.82)

LOA: limits of agreement; PE: percentage error; ICC: intraclass correlation coefficient; CI: confidence interval.

All ICCs for consistency and absolute agreement were significant at *P* < 0.001.

## Discussion

This initial validation study showed that the smart walker–integrated gait analysis system provides comparable data to the GAITRite system for the temporal gait parameters of stride, swing and stance time in potential smart walker users. Although also a good consistency for the stride length and gait speed was found between these two systems, they cannot be used interchangeably when absolute values of these spatial-related gait parameters are required (e.g. for comparison with normative values).

The low absolute agreement for spatial-related gait parameters can be explained by the fact that the GAITRite system refers to the distance between heel contacts on the electronic walkway for measuring the stride length, while the smart walker–integrated gait analysis system refers to the distance between leg placements recorded by the laser range finder 35 cm above the walkway. The reference points of the laser range finder are closer to the pivot of the lower legs (i.e. knee joint) and thus travel a shorter distance during the gait cycle, resulting in the shorter stride length and also the lower gait speed. While absolute agreement for these spatial-related parameters seems lacking, the extent to which they agree with the GAITRite system on the relative values (i.e. consistency) was good to excellent, suggesting that the stride length and gait speed of the smart walker–integrated gait analysis system may be themselves reliable and as good as those of the GAITRite system in determining meaningful changes in a user’s walking pattern.

Compared to previous validation studies of smart walker–integrated gait analysis systems, the strengths of this study are that a reasonable number of participants representative of potential smart walker users were recruited, a well-established, validated gait analysis system was used as criterion standard for comparison, and the data obtained were analysed by adequate statistical methods. However, this study also has some limitations. Only short straight walking in a controlled laboratory environment was evaluated, as limited by our criterion standard. Future studies should assess the validity of the smart walker-gait analysis system in less constrained movement situations. Our participants were predominantly females, limiting the generalizability of the results to males. However, we did not expect gender to affect the concurrent validity between the two systems.

The smart walker–integrated gait analysis system can provide clinicians and researchers the ability to unobtrusively capture gait parameters of smart walker users, without any sensors being attached to the user’s body. Our study represents a first step towards a continuous gait analysis of smart walker users in natural environments. The applicability of the system for such long-term gait monitoring needs to be confirmed in future studies.

**Clinical Messages**

- The smart walker–integrated gait analysis system has good concurrent validity with the GAITRite^®^ system for measuring temporal gait parameters in potential smart walker users.
- Stride length and gait speed can also be measured consistently; however, modifications are recommended to improve the absolute measurement accuracy for these spatial-related gait parameters.

## References

[1] Chen S Lach J Lo B, et al Toward pervasive gait analysis with wearable sensors: a systematic review. IEEE J Biomed Health Inform 2016; 20(6): 1521–1537. 28113185 10.1109/JBHI.2016.2608720

[2] Martins M Santos C Frizera A, et al A review of the functionalities of smart walkers. Med Eng Phys 2015; 37(10): 917–928. 26307456 10.1016/j.medengphy.2015.07.006

[3] Paulo J Peixoto P Nunes UJ. ISR-AIWALKER: Robotic walker for intuitive and safe mobility assistance and gait analysis. IEEE Trans Hum Mach Syst 2017; 47(6): 1110–1122.

[4] Wang T Merlet J-P Sacco G, et al Walking analysis of young and elderly people by using an intelligent walker ANG. Rob Auton Syst 2016; 75: 96–106.

[5] Ballesteros J Urdiales C Martinez AB, et al Automatic assessment of a rollator-user’s condition during rehabilitation using the i-Walker platform. IEEE Trans Neural Syst Rehabil Eng 2017; 25(11): 2009–2017. 28459694 10.1109/TNSRE.2017.2698005

[6] Werner C Moustris GP Tzafestas CS, et al User-oriented evaluation of a robotic rollator that provides navigation assistance in frail older adults with and without cognitive impairment. Gerontology 2018; 64(3): 278–290. 29183035 10.1159/000484663

[7] Guralnik JM Ferrucci L Pieper CF, et al Lower extremity function and subsequent disability: consistency across studies, predictive models, and value of gait speed alone compared with the short physical performance battery. J Gerontol A Biol Sci Med Sci 2000; 55(4): M221–M231. 10811152 10.1093/gerona/55.4.m221 PMC12149745

[8] Folstein MF Folstein SE McHugh PR. ‘Mini-mental state’. A practical method for grading the cognitive state of patients for the clinician. J Psychiatr Res 1975; 12(3): 189–198. 1202204 10.1016/0022-3956(75)90026-6

[9] Webster KE Wittwer JE Feller JA. Validity of the GAITRite walkway system for the measurement of averaged and individual step parameters of gait. Gait Posture 2005; 22(4): 317–321. 16274913 10.1016/j.gaitpost.2004.10.005

[10] Efthimiou E Fotinea S-E Goulas T, et al The MOBOT platform – showcasing multimodality in human-assistive robot interaction. In: Antona M Stephanidis C (eds) Universal access in human-computer interaction techniques and environments 10th international conference, UAHCI 2016, held as part of HCI International 2016, Toronto, ON, Canada, July 17–22, 2016, proceedings, part II. Berlin: Springer International Publishing, 2016, pp.382–391.

[11] Chalvatzaki G Papageorgiou XS Tzafestas CS. Towards a user-adaptive context-aware robotic walkerwith a pathological gait assessment system: first experimental study. In: 2017 IEEE/RSJ international conference on intelligent robots and systems (IROS), Vancouver, BC, Canada, 24–28 September 2017, pp.5037–5042. New York: IEEE.

[12] Bland JM Altman DG. Statistical methods for assessing agreement between two methods of clinical measurement. Lancet 1986; 1(8476): 307–310. 2868172

[13] Critchley LA Critchley JA. A meta-analysis of studies using bias and precision statistics to compare cardiac output measurement techniques. J Clin Monit Comput 1999; 15(2): 85–91. 12578081 10.1023/a:1009982611386

[14] Fleiss JL. The design and analysis of clinical experiments. New York: John Wiley & Sons, 1986.

[15] Zou GY. Sample size formulas for estimating intraclass correlation coefficients with precision and assurance. Stat Med 2012; 31(29): 3972–3981. 22764084 10.1002/sim.5466

